# Targeting IL-36 improves age-related coronary microcirculatory dysfunction and attenuates myocardial ischemia/reperfusion injury in mice

**DOI:** 10.1172/jci.insight.155236

**Published:** 2022-03-08

**Authors:** Juma El-Awaisi, Dean P.J. Kavanagh, Marco R. Rink, Chris J. Weston, Nigel E. Drury, Neena Kalia

**Affiliations:** 1 Microcirculation Research Group, Institute of Cardiovascular Sciences, and; 2 Institute of Immunology and Immunotherapy, College of Medical and Dental Sciences, University of Birmingham, Birmingham, United Kingdom.

**Keywords:** Aging, Inflammation, Cardiovascular disease, Microcirculation, Neutrophils

## Abstract

Following myocardial infarction (MI), elderly patients have a poorer prognosis than younger patients, which may be linked to increased coronary microvessel susceptibility to injury. Interleukin-36 (IL-36), a newly discovered proinflammatory member of the IL-1 superfamily, may mediate this injury, but its role in the injured heart is currently not known. We first demonstrated the presence of IL-36(α/β) and its receptor (IL-36R) in ischemia/reperfusion-injured (IR-injured) mouse hearts and, interestingly, noted that expression of both increased with aging. An intravital model for imaging the adult and aged IR-injured beating heart in real time in vivo was used to demonstrate heightened basal and injury-induced neutrophil recruitment, and poorer blood flow, in the aged coronary microcirculation when compared with adult hearts. An IL-36R antagonist (IL-36Ra) decreased neutrophil recruitment, improved blood flow, and reduced infarct size in both adult and aged mice. This may be mechanistically explained by attenuated endothelial oxidative damage and VCAM-1 expression in IL-36Ra–treated mice. Our findings of an enhanced age-related coronary microcirculatory dysfunction in reperfused hearts may explain the poorer outcomes in elderly patients following MI. Since targeting the IL-36/IL-36R pathway was vasculoprotective in aged hearts, it may potentially be a therapy for treating MI in the elderly population.

## Graphical abstract

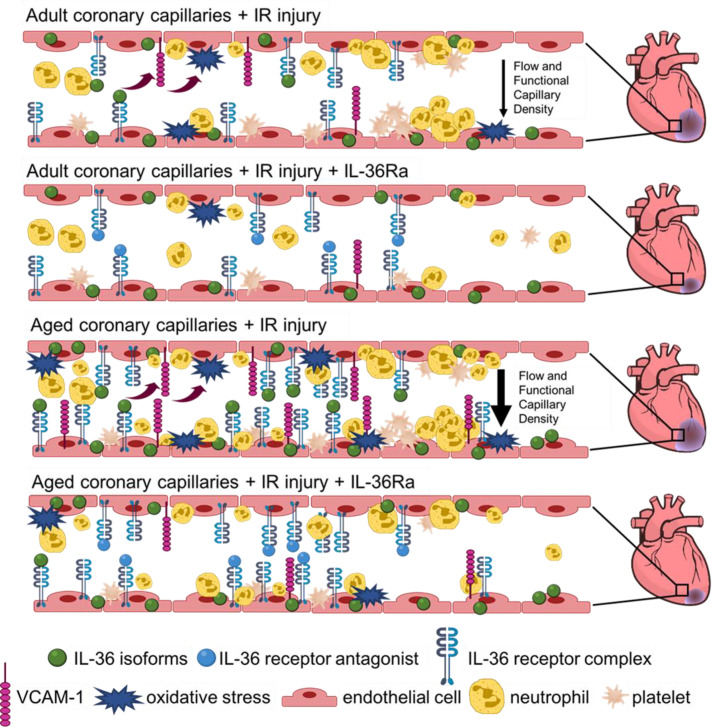

## Introduction

Treatment of ST-elevation myocardial infarction (MI) focuses on rapidly reestablishing perfusion following blockage in one or more of the epicardial coronary arteries. This can be achieved by primary percutaneous coronary intervention (PCI) using a coronary stent to open the culprit artery. Despite these interventions, a substantial proportion of patients still incur extensive muscle damage and develop heart failure after MI (1). This is partly due to reperfusion paradoxically leading to additional tissue damage, a condition termed ischemia/reperfusion (IR) injury. Indeed, restoration of normal epicardial blood vessel flow, but with suboptimal myocardial perfusion, can be observed in up to 50% of patients following PCI, leading to worse outcomes than in patients with full perfusion recovery (2). This suggests tissue damage likely occurs subsequent to inadequate coronary microcirculatory perfusion (3, 4). Microvascular involvement is further highlighted by observations that patients may present with MI but with a normal coronary angiogram (4). Increased clinical recognition of the importance of the coronary microcirculation has meant strategies to improve potential perturbations within it have gained recent attention (3, 4). However, current clinical tools cannot resolve microvessels less than 200 μm, and so little is known about the full range of cardiac microcirculatory responses to IR injury in vivo.

Age is a major risk factor for ischemic cardiovascular disease, increasing cardiac damage caused by IR injury, independent of “traditional” risk factors (5–7). Clinically, there is an increased incidence of heart failure, atrial fibrillation, and tachycardia in patients post-MI that increases with age (7). In experimental studies, coronary blood flow is much lower postreperfusion in senescent rats (8). Interestingly, the ability of the heart to respond to cardioprotective interventions is also lost with aging (8, 9). The term “inflammaging” describes the phenomenon of aging accompanied by a chronic low-grade, sterile inflammation that persists in the absence of an overt inflammatory stimulus (10). It is characterized by raised levels of proinflammatory cytokines that contribute to gradual tissue damage as well as altered responses to acute inflammatory injuries. The drivers underlying this persistent inflammation appear to involve increased reactive oxygen species (ROS) production in the vasculature, decreased antioxidative ability, and changes in the number, structure, and function of immune cells (11). In particular, increased neutrophil presence is detected in otherwise healthy but aged tissues. Neutrophil chemotaxis is also impaired, with serious implications as adherent cells cannot exit or transmigrate out of blood vessels (12). Here, they continue to release tissue-destructive proteases and ROS and cause vascular congestion as observed in aged, injured mouse lungs (13). Therefore, inflammaging may contribute to the enhanced age-related cardiovascular risk and poorer outcomes through actions on the local microcirculation. However, little is known in vivo about how age affects the coronary microcirculation in health and whether it increases the likelihood of microvascular disturbances after reperfusion injury.

The interleukin-1 family (IL-1F) consists of 11 known pro- and/or antiinflammatory cytokines, some of which have been studied extensively whereas others have received less attention (14). These are frequently the first and most upstream cytokines produced in response to injury and so are considered good targets for intervention (15). Since IL-1F members critically mediate sterile inflammation, they may be key mechanistic contributors causing myocardial microcirculatory disturbances postreperfusion (16). Indeed, the large-scale canakinumab antiinflammatory thrombosis outcomes study (CANTOS) trial provided exciting evidence that targeting this cytokine was beneficial in improving long-term outcomes post-MI in the absence of lipid lowering (17). In the last decade, genes encoding a novel cytokine cluster, namely interleukin-36 (IL-36), with structural and functional similarities to IL-1, were discovered (18, 19). IL-36, a collective name for 3 agonist ligands, IL-36α, IL-36β, and IL-36γ (previously called IL-1F6, IL-1F8, and IL-1F9), is fast emerging as a novel player regulating both innate and adaptive immune responses in a number of acute and chronic disorders. As well as amplifying IL-1 effects, IL-36 is also a potent mediator of inflammation in its own right. Indeed, its critical role in psoriasis, equaling if not surpassing that of IL-1, is well established, with roles in Crohn’s disease, airway infections, and rheumatoid arthritis recently identified (20–22).

IL-36 cytokines can be released from many sources, including epithelial cells, keratinocytes, fibroblasts, macrophages, monocytes, lymphocytes, and neurons, with neutrophils identified as an IL-36 source recently (18, 23–25). They signal through the IL-36 receptor (IL-36R), a heterodimer formed of IL-1Rrp2 and an accessory coreceptor protein, IL-1RacP (20). This receptor is also widely expressed at low levels in many organs and cell types, including leukocytes and vascular endothelial cells. Downstream intracellular signaling leads to NF-κB and MAPK activation and subsequent secretion of multiple potent proinflammatory mediators, including TNF-α, IL-1β, IL-6, and IL-8. The actions of the 3 agonists can be inhibited by a naturally occurring antagonist, namely the IL-36 receptor antagonist (IL-36Ra) (22–25). Although we know the IL-36/IL36R pathway is highly proinflammatory in the skin and lungs, we are still at a very early stage in our current understanding of its in vivo biology, and no previous studies to our knowledge have investigated a role for this pathway in mediating inflammation in the IR-injured heart. Its downstream transcription factor, NF-κB, is a known mediator of coronary microvascular injury as inhibition of NF-κB in 2 independent rabbit models of myocardial IR injury reduced inflammation and the no-reflow area (26, 27). Therefore, the IL-36 signaling pathway is a potential target for cardioprotective interventions.

We have previously described an intravital imaging technique that allows the microcirculation of the anesthetized mouse beating heart to be imaged with cellular resolution in vivo (28). Using this technique, we have shown that despite deceptive hyperemic responses, increased microcirculatory flow heterogeneity, reduction in functional capillary density, and a marked thromboinflammatory infiltration were present within coronary capillaries immediately postreperfusion. The current study used this imaging model to determine whether the clinically observed age-related poorer outcomes post-MI were related to an increased susceptibility of the aged coronary microcirculation to myocardial IR injury. The presence of IL-36 and its receptor was investigated in the heart and the impact of age on their expression determined. To ascertain whether IL-36 could mediate coronary microcirculatory perturbations, the ability of IL-36 agonists to directly induce inflammation in the beating heart was imaged, and the effects of treatment with the IL-36Ra were determined. Mechanistic insights into how IL-36Ra may confer vasculoprotection via attenuating ROS-mediated endothelial oxidative damage and VCAM-1 expression are also presented.

## Results

### Age increases expression of IL-36R in healthy and IR-injured hearts, which was also induced in endothelial cells stimulated with TNF-α or IL-36 cytokines.

**Figure 1:**
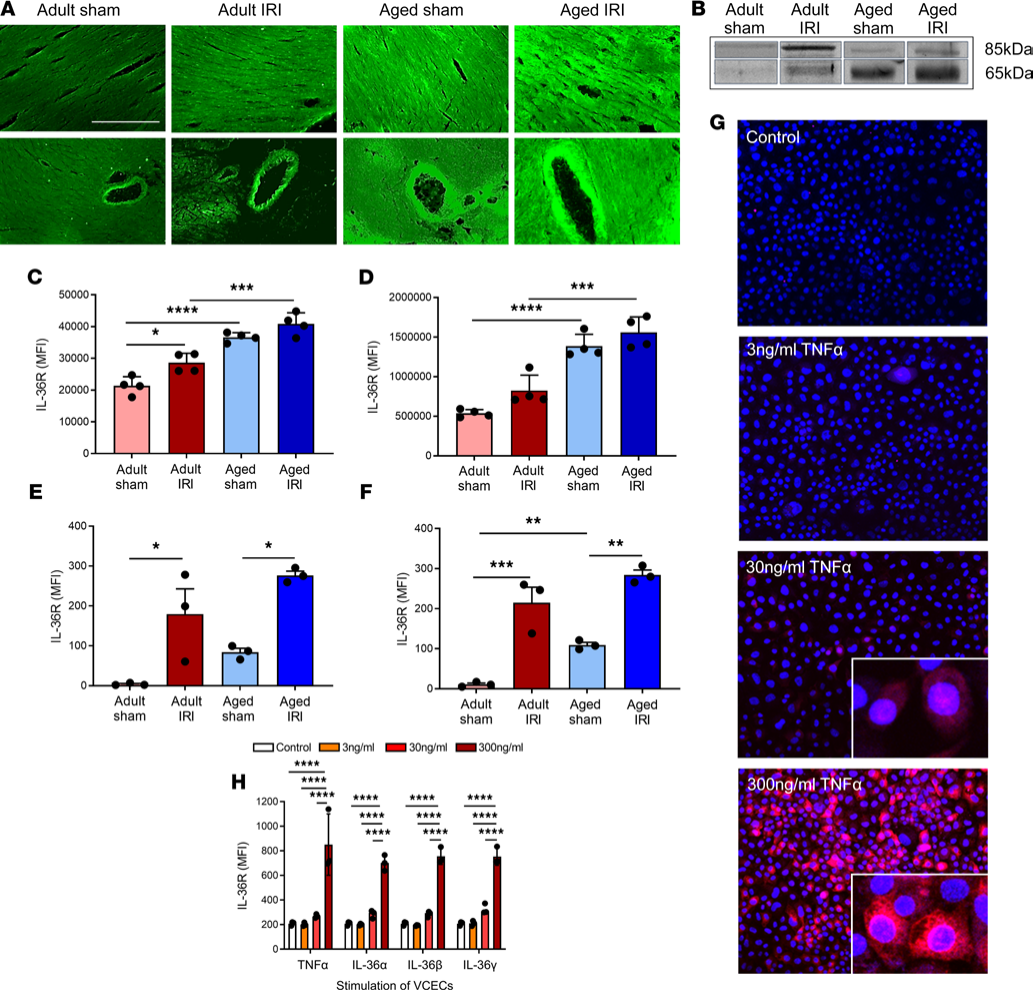
Age increases expression of IL-36R in healthy and IR-injured hearts, and expression can also be induced in endothelial cells with TNF-α or IL-36 cytokine stimulation. Hearts were assessed for IL-36R using immunostaining, Western blot analysis, and flow cytometry. Representative images of IL-36R (green) staining of (**A**) frozen heart sections and (**B**) Western blots. The molecular weight of IL-36R is about 65 kDa, but it migrates to the position of about 85 kDa in denaturing protein gels. Hence, 2 bands were observed corresponding to 65 kDa, the more active protein due to cleavage of its signaling peptide, and 85 kDa, the less potent glycosylated form. Quantitative analysis of the (**C**) immunofluorescence images (*n* = 4/group) and (**D**) Western blots (*n* = 4/group). *P* = 0.0146 adult sham vs. adult IR immunofluorescence. *P* = 0.0003 aged sham vs. aged IR immunofluorescence. *P* = 0.001 aged sham vs. aged IR Western blot. Hearts were also collagenase digested and analyzed flow cytometrically for IL-36R expression. Flow cytometry analysis confirmed that IR injury and age induced a significant increase in IL-36R on (**E**) coronary endothelial cells and (**F**) cardiomyocytes (*n* = 3/group). (**G** and **H**) Murine vena cava endothelial cells (VCECs) were cultured and stimulated for 4 hours with experimental media (control), an IL-36 cytokine (α, β, or γ), or TNF-α. (**G**) Representative images of IL-36R (red) expression on stimulated, nonpermeabilized cells (Hoechst 33342–stained nuclei in blue). (**H**) Quantitative analysis of IL-36R expression on VCECs following stimulation (*n* = 3/group). Scale bar indicates 200 μm. *P < 0.05, **P < 0.01, ***P < 0.001, ****P < 0.0001 as determined using a 1-way ANOVA followed by a Tukey’s post hoc test.

Immunofluorescence staining of frozen heart sections demonstrated very low constitutive expression of IL-36R in adult sham-injured hearts. This increased (*P* = 0.0146) in response to IR injury in adult hearts. Basal expression of IL-36R was higher (*P* < 0.0001) in aged sham hearts when compared with adult shams. This was further increased (*P* = 0.0003) in aged injured hearts when compared with adult injured hearts (Figure 1, A and C). Western blot analysis showed similar stepwise increases in the presence of the IL-36R protein. The molecular weight of IL-36R is approximately 65 kDa, but it migrates to the position of approximately 85 kDa in denaturing protein gels (29). Hence, 2 bands were observed corresponding to a 65 kDa, active protein, due to cleavage of its signaling peptide, and an 85 kDa, less potent, glycosylated form (Figure 1B). Again, adult sham hearts expressed very low levels of IL-36R. This increased in adult injured hearts, which expressed the highest levels of the 85 kDa protein. However, significant increases (*P* < 0.0001) in IL-36R protein were expressed in aged sham hearts when compared with adult sham hearts, particularly of the more potent 65 kDa protein. This expression increased (*P* = 0.001) further in aged injured hearts, which exhibited the highest levels of the active 65 kDa IL-36R protein (Figure 1, B and D). To further confirm, at a more cellular level, the expression of IL-36R on coronary endothelial cells, hearts were collagenase digested and flow cytometrically analyzed. Very low basal levels of IL-36R were noted on adult sham coronary endothelial cells, but IL-36R was significantly (*P* < 0.05) increased on endothelial cells from adult IR-injured hearts. Aged sham coronary endothelial cells again showed higher IL-36R than on adult sham endothelial cells, which was also significantly (*P* < 0.05) increased with injury (Figure 1E). Interestingly, a similar pattern of IL-36R expression was noted flow cytometrically on cardiomyocytes (Figure 1F). To further identify whether IL-36R was expressed specifically on endothelial cells, murine vena cava endothelial cells (VCECs) were stained for IL-36R. Constitutive expression of IL-36R on unstimulated VCECs was very low. However, stimulation of VCECs with TNF-α, as well as IL-36 isoforms, significantly (*P* < 0.0001) upregulated IL-36R expression, with no differences observed between cytokine treatments (Figure 1, G and H).

### Age increases expression of IL-36α, IL-36β, and VCAM-1 in healthy and IR-injured hearts.

**Figure 2:**
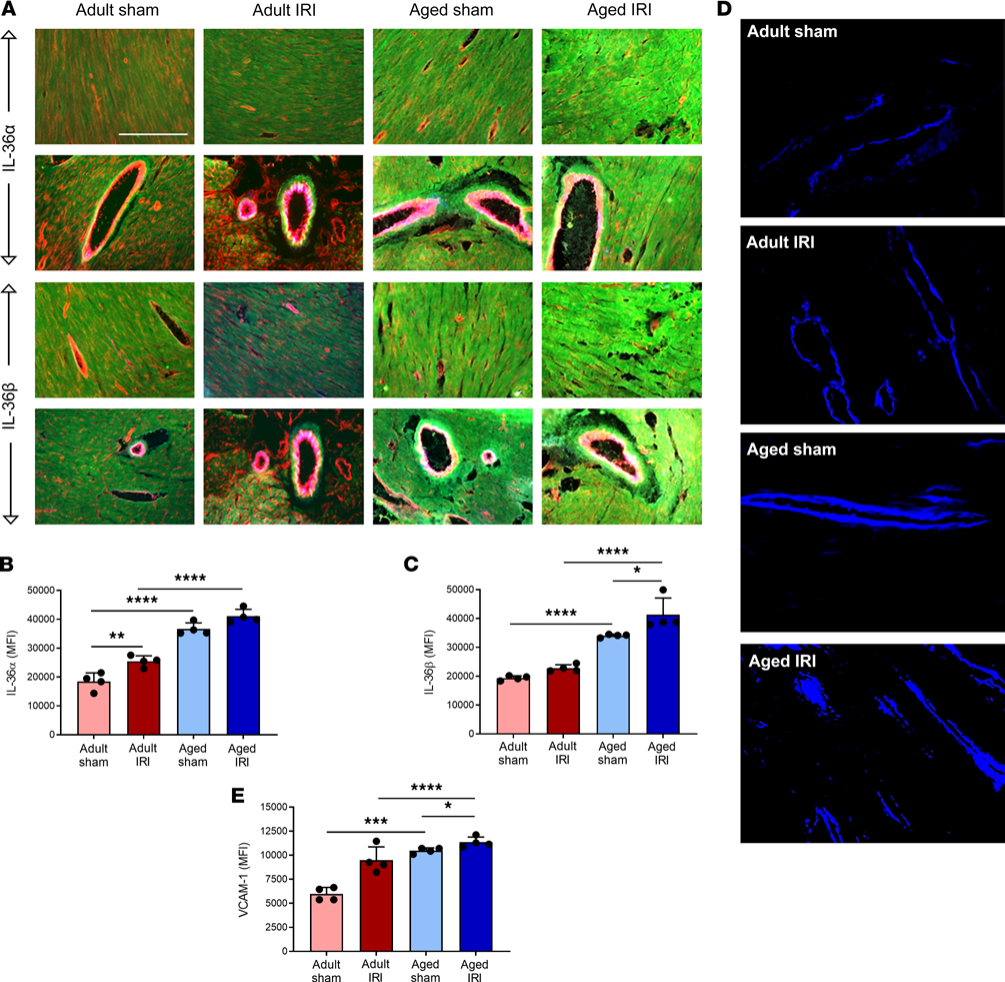
Age increases expression of IL-36α, IL-36β, and VCAM-1 in healthy and IR-injured hearts. Hearts from adult and aged sham and IR-injured mice were immunostained with an anti–IL-36α/β, anti-CD31, and anti–VCAM-1 antibody. (**A**) Representative images of IL-36α (green; top 2 rows) and IL-36β (green; bottom 2 rows) staining of frozen heart sections costained with CD31 (red) and VCAM-1 (blue). The upper row of each cytokine panel shows coronary microvessels with the lower row selected to demonstrate staining of a large coronary blood vessel. Quantitative analysis of the immunofluorescence images for (**B**) IL-36α and (**C**) IL-36β expression. *P = 0.0193. **P = 0.0054. ****P < 0.0001. (**D**) Representative images of VCAM-1 (blue) staining of frozen heart sections. (**E**) Quantitative analysis of immunofluorescence images of VCAM-1 expression. *P = 0.0308. ***P = 0.0002. ****P < 0.0001. Scale bar indicates 200 μm. *n* = 4/group. *P* values were determined using a 1-way ANOVA followed by a Tukey’s post hoc test.

Immunofluorescence staining for IL-36α and IL-36β revealed a similar pattern of expression in the heart to the IL-36R (Figure 2, A–C). Expression of the endothelial surface adhesion molecule, VCAM-1, was also investigated to better understand the impact of age on a well-established inflammatory marker. VCAM-1 was expressed on the larger vasculature of the heart rather than on coronary capillaries in all 4 groups and significantly increased in a stepwise manner with injury and age (Figure 2, D and E).

### Inducible expression of IL-36R and IL-36 cytokines occurs on coronary microvasculature.

**Figure 3:**
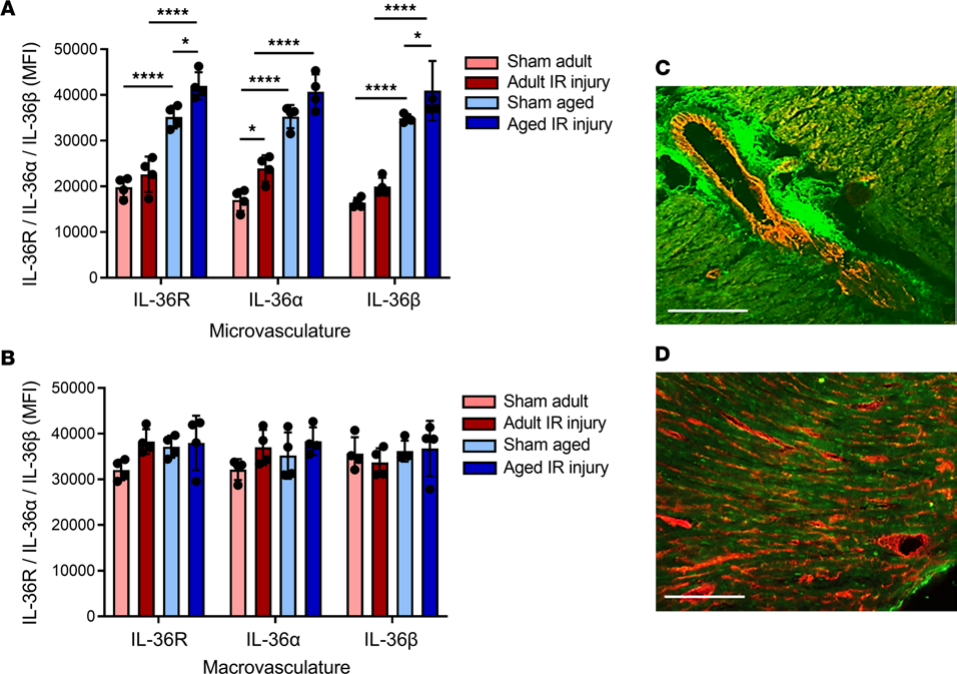
Changes in the expression of IL-36 cytokines and its receptor occur on the coronary microvasculature and not on the large blood vessels. Quantitative analysis of IL-36R, IL-36α, and IL-36β expression using immunofluorescence is shown for the (**A**) microvasculature and (**B**) macrovasculature of the adult and aged sham and IR-injured heart. *n* = 4/group. *P < 0.05, ****P < 0.0001 as determined using a 1-way ANOVA followed by a Tukey’s post hoc test. To further determine whether IL-36R (green) expression was vascular in nature, heart sections were costained with an anti-CD31 antibody (red) and imaged using a multiphoton microscope. Representative image of (**C**) coronary macrovasculature and (**D**) microvasculature for an adult IR-injured mouse.

Having demonstrated an increased vascular expression of IL-36R and IL-36 cytokines with IR injury and age, we further examined which component of the vascular network this increase occurred on by comparing the mean fluorescence intensity (MFI) between microvessels (capillaries) and macrovessels. Expression of IL-36R, IL-36α, and IL-36β significantly increased with age and IR injury specifically on the microvasculature (Figure 3A). In contrast, expression on the macrovasculature remained relatively constant in all 4 groups (Figure 3B).

To further confirm that IL-36R expression was indeed present on blood vessels, heart sections were costained with an anti-CD31 antibody. IL-36R expression was intense within the wall of some of the larger blood vessels, particularly in the tunica adventitial layer, as well as on the coronary capillaries (Figure 3, C and D).

### Topical application of IL-36 is proinflammatory in adult and aged beating hearts in vivo.

**Figure 4:**
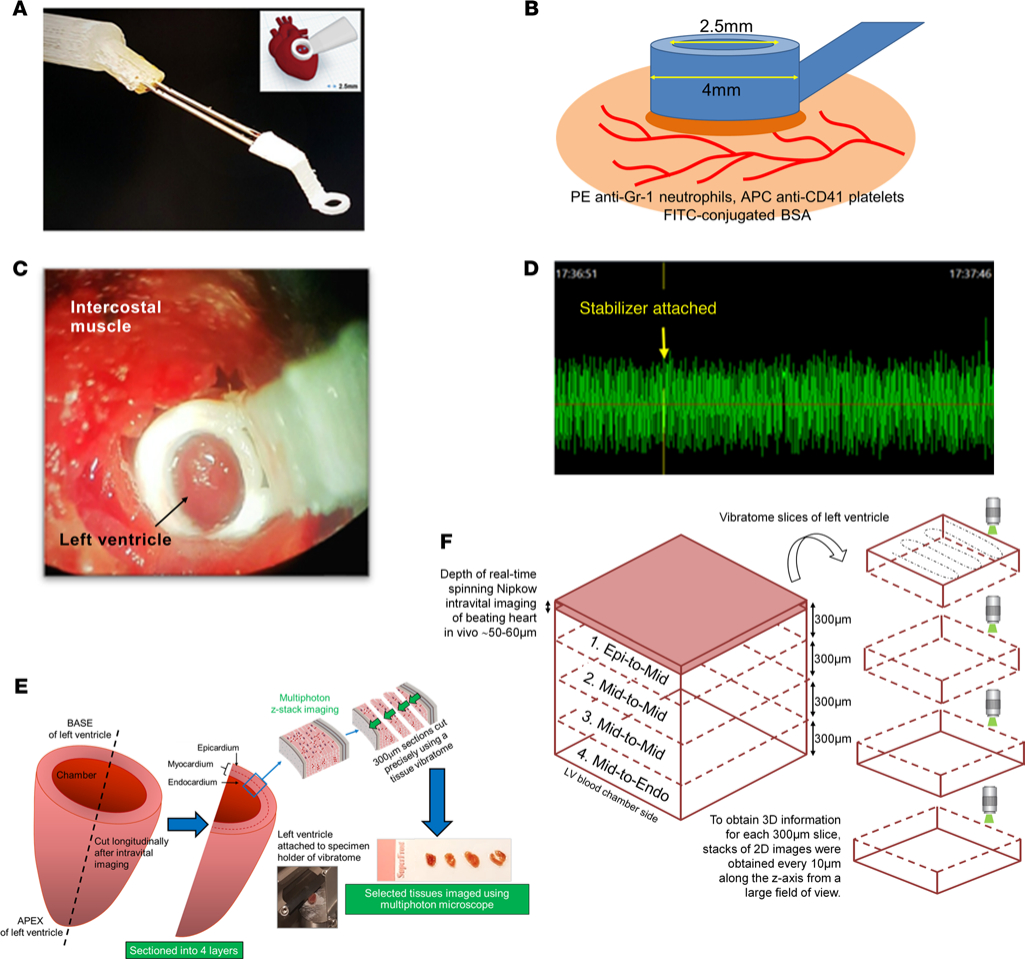
Intravital imaging of the mouse beating heart microcirculation in vivo and multiphoton imaging of the heart ex vivo. (**A**–**C**) An in-house–designed 3D-printed stabilizer is lowered onto the beating left ventricle, allowing confocal intravital imaging in its center. Only a small surface of the beating heart has its motion reduced enough to permit imaging. (**D**) No BP/heart rate changes were detected using this approach as determined by photoplethysmography in both adult and aged sham and IR-injured mice. The graph presented shows BP, which remains constant even after the stabilizer is attached. (**E**) To ascertain whether any thromboinflammatory and vasculoprotective events imaged intravitally on the surface of the heart were also occurring in the deeper layers of the myocardium, multiphoton microscopy was used. The heart was cut in half longitudinally from the base to apex to expose the inner endocardial layer lining the left ventricle chamber. It was then placed on a specimen holder and attached to a tissue vibratome to precisely section the left ventricle wall into 4 × 300 μm thickness sections from the outermost layer closest to the epicardium, through to the inner layer closest to the endocardium. (**F**) Multiphoton *Z*-stacks were taken from all 4 layers, namely the (i) outermost layer closest to the epicardium — epi-to-mid (ii) outer myocardial layer — mid-to-mid (iii) inner myocardial layer — mid-to-mid, and (iv) innermost layer closest to the endocardium — mid-to-endo, avoiding the last section if it had “missing” myocardium due to sectioning through the actual ventricle chamber. Images from each layer were then rendered to form 3D stack images.

**Figure 5:**
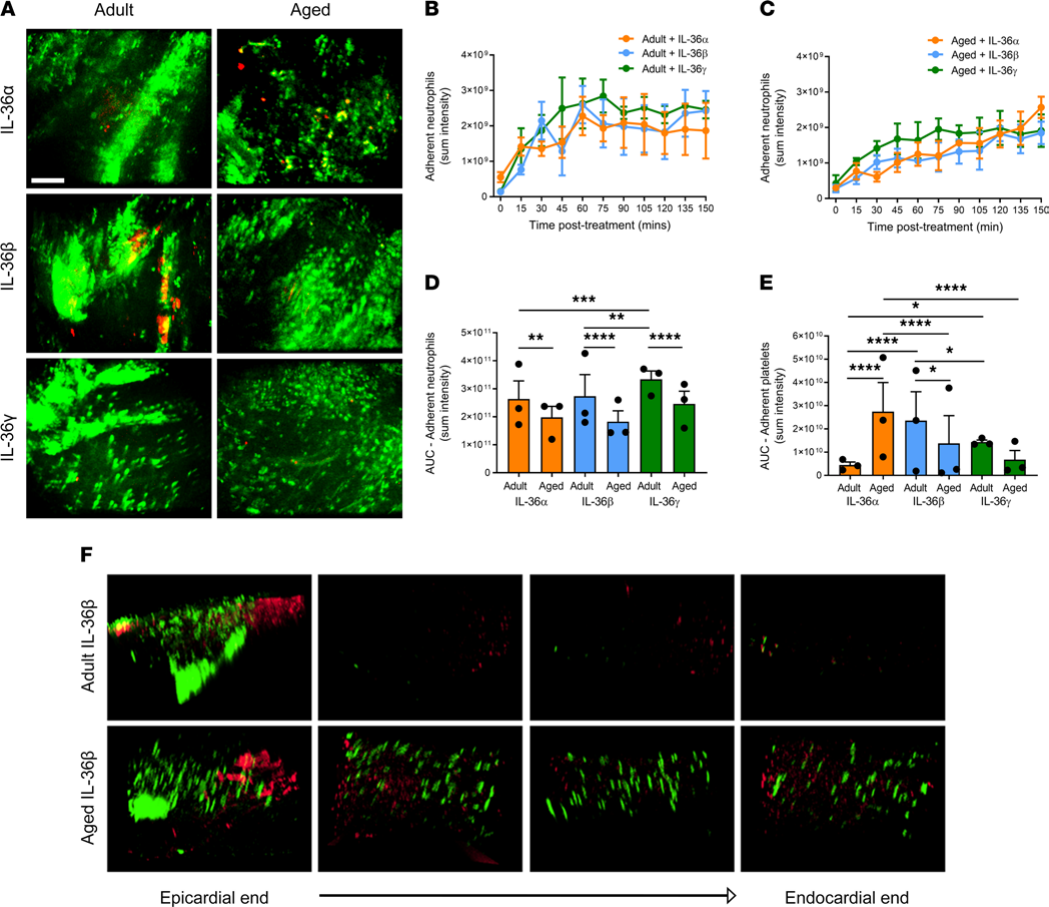
Topically applied IL-36 is proinflammatory in the adult and aged beating heart in vivo. IL-36α, IL-36β, or IL-36γ (200 ng/mL) was topically applied to the healthy adult and aged beating heart left ventricle. (**A**) Representative intravital images of the beating heart showing adherent neutrophils (green) and platelets (red) in the coronary microcirculation at 120 minutes postapplication. Quantitative analysis of the intravital data for adherent neutrophils for the (**B**) adult and (**C**) aged groups and the area under the curve (AUC) for (**D**) adherent neutrophils and (**E**) platelets over a time course of 150 minutes. (**F**) Representative multiphoton images of neutrophils (green) and platelets (red) in all 4 layers of the left ventricle exposed to IL-36β in adult (top row) and aged (lower row) mice. Images are taken from the outermost layer closest to the epicardium, outer myocardial layer, inner myocardial layer, and innermost layer closest to the endocardium. Scale bar indicates 100 μm. *n* = 3/group. *P < 0.05, **P < 0.01, ***P < 0.001, ****P < 0.0001 as determined using a 1-way ANOVA followed by a Tukey’s post hoc test.

To determine whether the IL-36R expressed in the heart was functional in vivo, intravital microscopy was used to directly visualize the ability of topical IL-36 to elicit an inflammatory response within the healthy adult and aged beating hearts. Intravital imaging of the beating heart was successfully achieved in anesthetized mice by attaching a 3D-printed stabilizer to the left ventricle (Figure 4, A–D). This reduced motion in the *x*-*y* plane of a small region of the ventricle sufficiently enough to permit imaging. All 3 IL-36 agonists were able to increase neutrophil recruitment to a similar degree in healthy adult hearts, although IL-36γ was slightly more potent (Supplemental Video 1; supplemental material available online with this article; https://doi.org/10.1172/jci.insight.155236DS1). This proinflammatory response was rapid and increased with time until a plateau was reached at approximately 60 minutes. In addition to neutrophil adhesion within coronary capillaries, a remarkable involvement of more medium-sized blood vessels was also observed. Indeed, these vessels were not usually visible but were clearly identified when delineated with adherent neutrophils. Platelet aggregates were occasionally observed in some of these medium-sized vessels, particularly in response to IL-36β and IL-36γ. These were mostly present in vessels where extensive clusters of adherent neutrophils were observed (Figure 5, A, B, D, and E). A similar statistically significant proinflammatory response was also observed when cytokines were topically applied to the healthy aged hearts, although levels were slightly lower and the response slower than that observed in adult mice. However, unlike in adult hearts where the response plateaued, in aged hearts exposed to IL-36α and IL-36β, neutrophil recruitment continued to rise. Platelet aggregates were again occasionally observed, but no significant differences were observed other than a decrease in their presence with IL-36β/γ (Figure 5, A and C–E). The number of free-flowing neutrophils generally decreased in response to all 3 IL-36 cytokine treatments in both adult and aged hearts (data not presented).

To ascertain whether thromboinflammatory events imaged intravitally on the surface of the heart were also occurring in the deeper layers of the myocardium, multiphoton microscopy was used. A vibratome was used to precisely section the left ventricle wall into 4 sections of 300 μm thickness from the outermost layer closest to the epicardium through to the inner layer closest to the endocardium. Multiphoton *Z*-stacks were taken from all 4 layers (Figure 4, E and F). The data obtained confirmed the ability of IL-36 cytokines to mediate a proinflammatory response in topically treated adult and aged hearts. However, this was only noted in the outermost layer of the heart muscle exposed to the topically applied cytokine and did not extend into the depths of the muscle wall, likely explained by the inability of the cytokine to permeate during the exposure period beyond the superficial myocardial layers. Multiphoton studies also detected a high basal presence of adherent neutrophils in aged mice throughout the muscle wall (Figure 5F).

### Age increases thromboinflammatory disturbances within healthy and IR-injured coronary microcirculation in vivo, which are prevented in both age groups by IL-36R inhibition.

**Figure 6:**
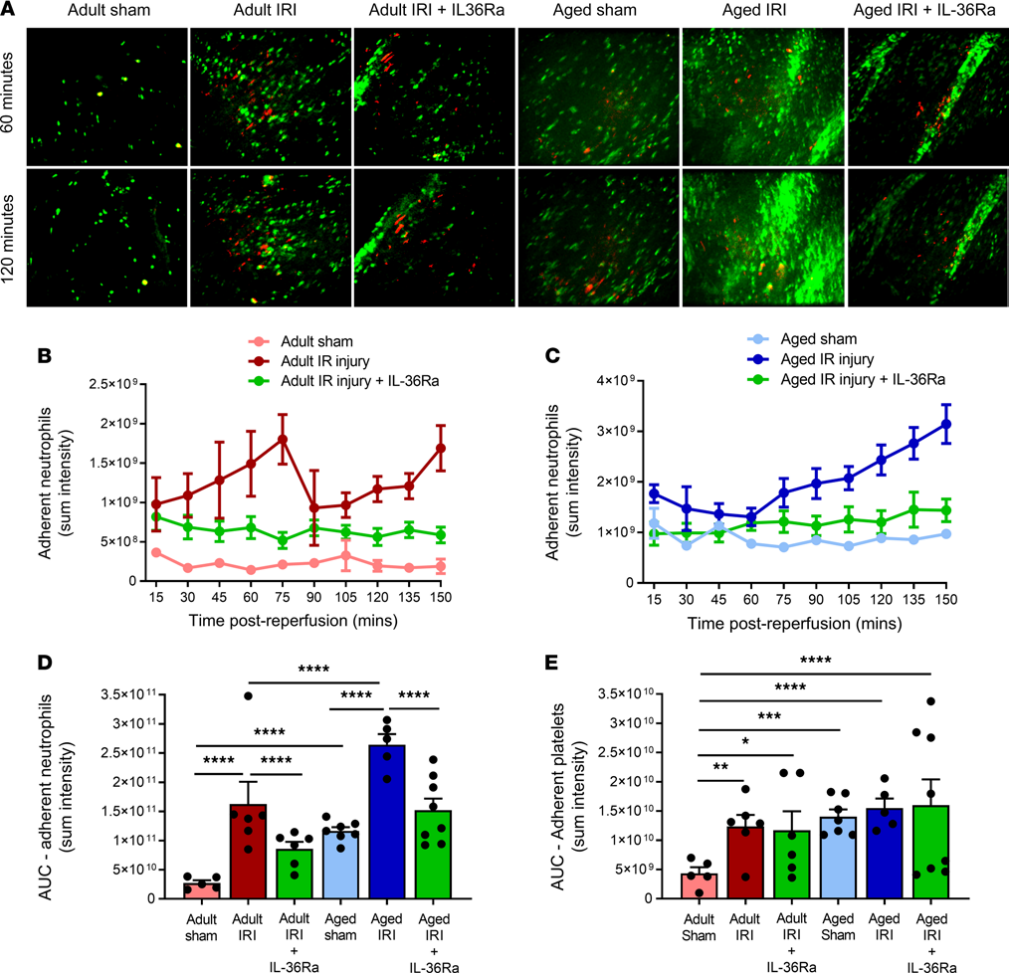
Age increases thromboinflammatory disturbances within the healthy and IR-injured beating heart coronary microcirculation in vivo, which can be prevented in both age groups by IL-36R inhibition. An IL-36Ra (15 μg/mouse) was injected intra-arterially at 10 minutes prereperfusion and 60 minutes postreperfusion in adult and aged mice. (**A**) Representative intravital images of the beating heart showing adherent neutrophils (green) and platelets (red) in the coronary microcirculation at 60 and 120 minutes in sham hearts or 60 and 120 minutes postreperfusion in injured hearts. Adherent neutrophils (green) and platelet microthrombi (red) are primarily within coronary capillaries in injured hearts. Intensely fluorescent green areas in aged IR-injured hearts correspond to medium-sized blood vessels that have become delineated by the presence of a substantial number of adherent neutrophils. Quantitative analysis of the intravital data for adherent neutrophils in (**B**) adult and (**C**) aged IR-injured hearts and the AUC for (**D**) adherent neutrophils and (**E**) platelets over a time course of 150 minutes. Scale bar indicates 100 μm. Adult sham — *n* = 5/group; adult IRI — *n* = 6/group; adult IRI + IL-36Ra — *n* = 5/group; aged sham — *n* = 7/group; aged IRI — *n* = 5/group; aged IRI + IL-36Ra — *n* = 8/group. *P = 0.0339, **P = 0.0014, ***P = 0.0003, ****P < 0.0001 as determined using a 1-way ANOVA followed by a Tukey’s post hoc test.

Intravital imaging demonstrated an increase (*P* < 0.0001) in adherent neutrophils, primarily within coronary capillaries, in response to IR injury in adult mice when compared with sham adult hearts (Supplemental Video 2). Basal neutrophil adhesion was also increased (*P* < 0.0001) in aged sham hearts when compared with adult shams. Neutrophil recruitment was greatest in aged IR-injured hearts, which was higher (*P* < 0.0001) than adult IR-injured hearts. This increase was noted at all time points postreperfusion and continued to rise. Individual neutrophils were often difficult to demarcate and appeared as clusters in aged injured hearts. Their presence was also noted in medium-sized coronary microvessels as well as in capillaries, something not observed in adult injured hearts (Figure 6, A–D). The number of free-flowing neutrophils decreased (*P* < 0.0001—data not presented) in all groups when compared with adult sham hearts with a concomitant and significant increase in the presence of small aggregates of platelets within coronary capillaries (Figure 6, A and E).

Intravital imaging further demonstrated the ability of exogenous IL-36Ra treatment to reduce neutrophil adhesion in both adult (*P* < 0.0001) and aged IR-injured hearts (*P* < 0.0001) when compared with their respective nontreated IR-injured groups (Figure 6, A–D). In some aged mice, neutrophil adhesion could still occasionally be observed within the medium-sized coronary vessels, but this was much lower compared with nontreated groups (Figure 6A). There was no statistically significant impact of IL-36Ra treatment on the presence of platelet microthrombi within adult or aged IR-injured hearts (Figure 6E).

### Age increases neutrophil presence within the deeper layers of the healthy and IR-injured myocardium.

**Figure 7:**
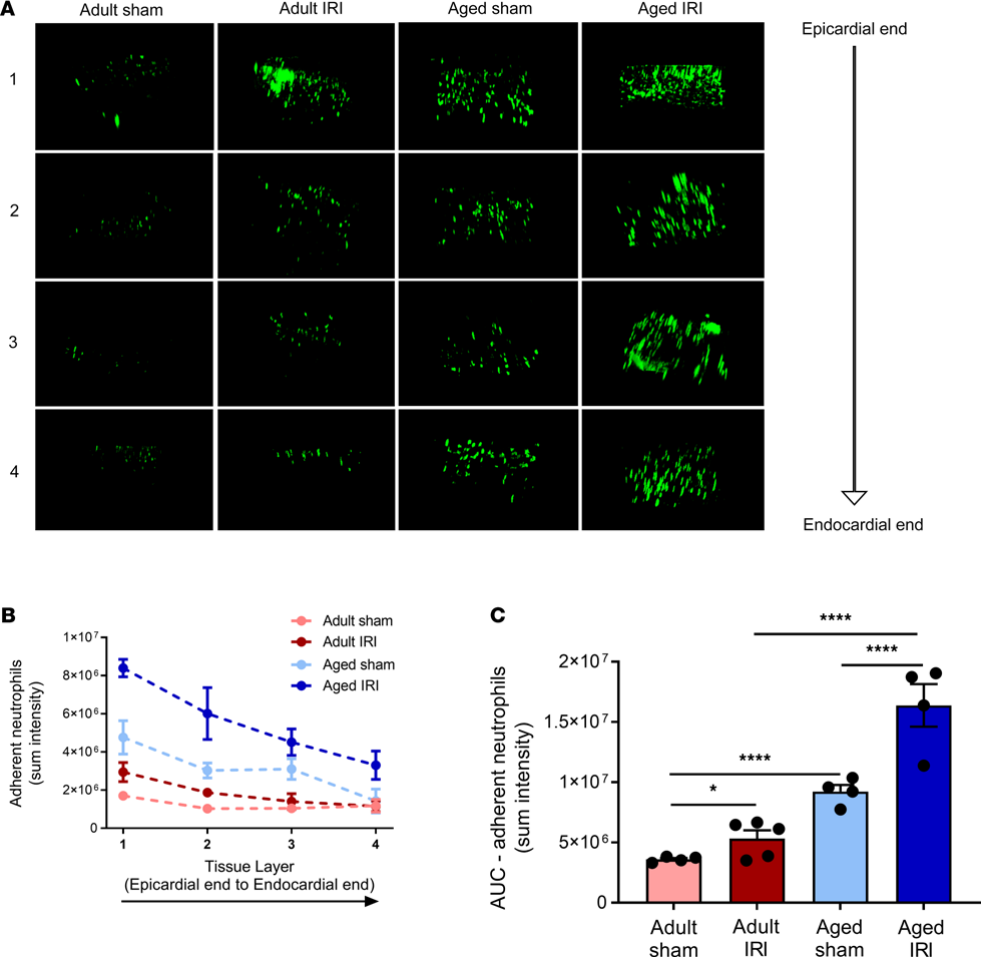
Age increases neutrophil presence within the deeper layers of the healthy and IR-injured myocardium. (**A**) The left ventricle was vibratome sectioned into four 300 μm sections and imaged using a multiphoton microscope. Representative *Z*-stack multiphoton images of neutrophils (green) in the 4 layers of the left ventricle taken from the outermost layer closest to the epicardium (first row), outer myocardial layer (second row), inner myocardial layer (third row), and innermost layer closest to the endocardium (fourth row). Quantitative analysis of the multiphoton data at various depths for (**B**) adherent neutrophils and corresponding (**C**) AUC for adherent neutrophils. Adult sham — *n* = 4/group; adult IRI — *n* = 5/group; aged sham — *n* = 4/group; aged IRI — *n* = 4/group. *P = 0.0325, ****P < 0.0001 as determined using a 1-way ANOVA followed by a Tukey’s post hoc test.

Minimal neutrophil presence was identified in adult sham hearts throughout the depth of the left ventricular wall when imaged ex vivo using multiphoton microscopy. However, in adult injured hearts, an increased (*P* = 0.035) presence of neutrophils was observed in all 4 layers of the heart when compared with adult sham hearts (Supplemental Video 3). However, the largest neutrophil presence in response to injury occurred within the outermost 300 μm layer. Basal neutrophil presence was also uniformly increased (*P* < 0.0001) throughout all 4 layers of the ventricle in aged sham hearts when compared with adult sham hearts (also seen in Figure 5F). This was further increased (*P* < 0.0001) in aged injured hearts when compared with adult injured hearts, with the greatest presence again noted within the outermost layer of the ventricle wall (Figure 7, A–C).

### IL-36R inhibition improved functional capillary density and reduced infarct size in vivo in adult and aged IR-injured hearts.

**Figure 8:**
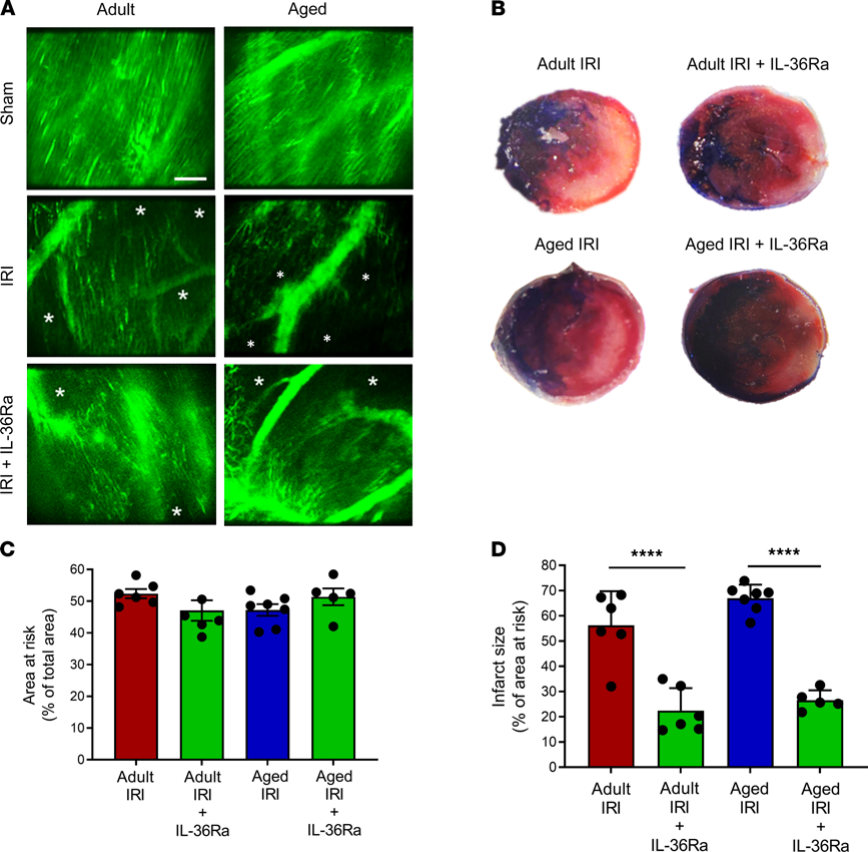
IL-36R inhibition improves functional capillary density and reduces infarct size in vivo in the IR-injured adult and aged heart. An IL-36Ra (15 μg/mouse) was injected intra-arterially at 10 minutes prereperfusion and 60 minutes postreperfusion in adult and aged mice. (**A**) Representative intravital images of FITC-BSA–perfused (green) coronary microvessels at 150 minutes in sham hearts or 150 minutes postreperfusion in injured hearts. *Areas not perfused with FITC-BSA. Scale bar indicates 100 μm. (**B**) Representative images of the TTC-stained infarct size in all 4 groups. (**C**) Quantitative analysis of the area at risk in all 4 groups. (**D**) Quantitative analysis of infarct size in all 4 groups. *n* = 6/group (*n* = 5 for aged IRI + IL-36Ra group). ****P < 0.0001 as determined using a 1-way ANOVA followed by a Tukey’s post hoc test. TTC, 2,3,5-triphenyltetrazolium chloride.

An extensive network of FITC-BSA–perfused capillaries was observed in both adult and aged sham mice, paralleling the arrangement of muscle fibers, with cross connections along their length. Focusing up and down on the field of view showed no areas devoid of perfused capillaries. Well-perfused medium-sized vessels were also visible in some fields of view. In contrast, IR injury of adult hearts was associated with multiple areas in which FITC-BSA did not perfuse. This resulted in patchy areas that appeared devoid of any microvasculature, indicating reduced functional capillary density. This was further reduced in aged IR-injured hearts. Indeed, in some fields of view, up to half of the imaged area appeared to lack perfusion. Of note, medium-sized vessels were still readily visible and well perfused in both adult and aged injured hearts. The use of an IL-36Ra was able to improve functional capillary density in both adult and aged hearts, although some areas of no perfusion were still visible (Figure 8A).

Infarct size appeared slightly larger in aged mice when compared with adult mice, but this was not significant. However, a significant decrease in infarct size was observed in both adult (*P* < 0.0001) and aged IR-injured hearts (*P* < 0.0001) receiving IL-36Ra treatment when compared with their respective nontreated IR-injured groups. The area at risk (AAR) (and nonischemic area) was not significantly different across all 4 groups (Figure 8, B–D).

### IL-36R inhibition reduced endothelial and cardiomyocyte oxidative damage and VCAM-1 expression in the IR-injured adult and aged heart.

**Figure 9:**
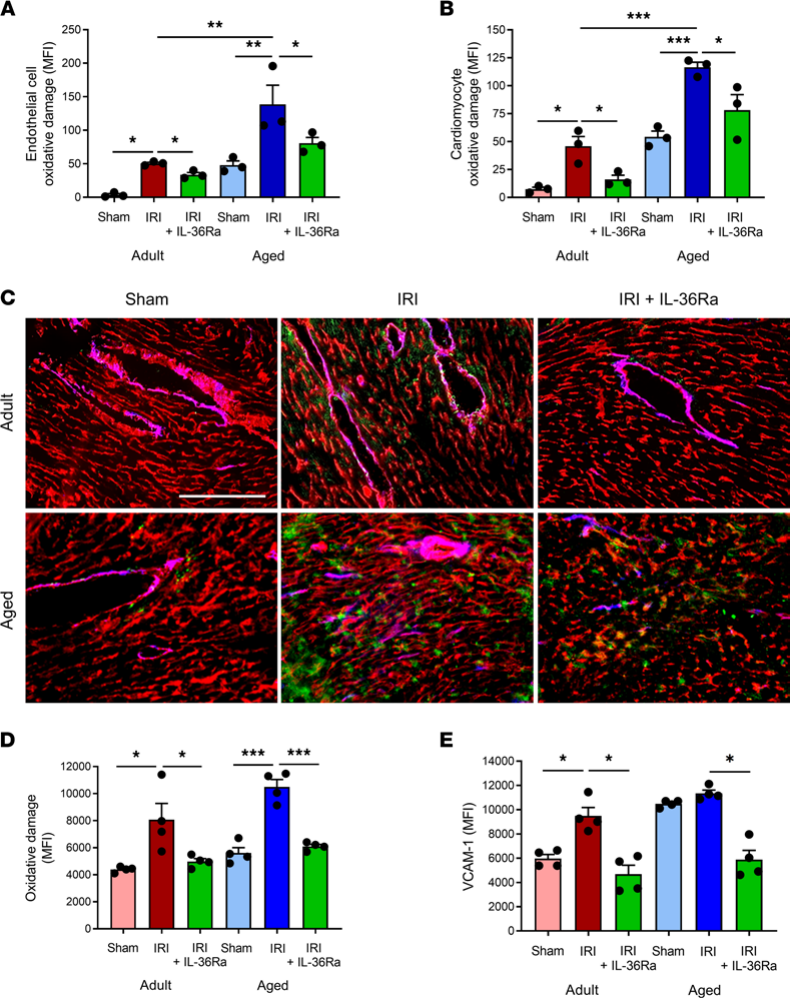
IL-36R inhibition reduces endothelial and cardiomyocyte oxidative damage and VCAM-1 expression in the IR-injured adult and aged hearts. Hearts from adult and aged sham, IR-injured, and IR-injured + IL-36Ra mice were either collagenase digested and analyzed flow cytometrically or sectioned and analyzed using immunofluorescence for oxidative damage and/or VCAM-1 expression. Flow cytometry analysis demonstrated that IR injury increased oxidative damage of both (**A**) adult and aged coronary endothelial cells and (**B**) adult and aged cardiomyocytes when compared with appropriate age sham cells. This was reduced in both cell populations in adult and aged mice treated with the IL-36Ra. *n* = 3/group. (**C**) Representative immunofluorescence images of CD31 (red) costained with DNA/RNA oxidative damage (green) and VCAM-1 (blue) in sham, IR-injured, and IL-36Ra–treated adult and aged mice. Quantitative analysis of the immunofluorescence images for myocardial (**D**) oxidative damage and (**E**) VCAM-1 expression. Scale bar indicates 200 μm. *n* = 4/group. *P < 0.05, **P < 0.01, ***P < 0.001 as determined using a 1-way ANOVA followed by a Tukey’s post hoc test.

To determine whether IL-36Ra treatment conferred vasculoprotection via mechanisms involving attenuation of endothelial oxidative stress and VCAM-1 expression, flow cytometry of collagenase-digested adult and aged hearts and immunofluorescence on frozen adult and aged heart sections were performed. IR injury increased ROS-mediated oxidative damage on both adult (*P* < 0.05) and aged (*P* < 0.01) coronary endothelial cells and adult (*P* < 0.05) and aged (*P* < 0.001) cardiomyocytes when compared with appropriate age sham heart cells as determined by flow cytometry. The damage was significantly greater in IR-injured aged hearts compared with IR-injured adult hearts. However, this damage was significantly (*P* < 0.05) reduced on both cell populations in all mice treated with the IL-36Ra regardless of age (Figure 9, A and B).

Significant oxidative damage in IR-injured adult (*P* < 0.05) and aged (*P* < 0.001) hearts was confirmed using immunofluorescence and was noted as punctate staining on both CD31^+^ vascular and nonvascular structures (Figure 9C). Moreover, this damage was noticeably greater on aged IR-injured hearts. This was again reduced in all mice treated with the IL-36Ra regardless of age (Figure 9D). VCAM-1 was also increased in adult (*P* < 0.05) IR-injured tissue, particularly around larger coronary blood vessels. In aged mice, basal VCAM-1 expression was high and did not increase further with IR injury. However, expression of this endothelial adhesion molecule decreased in both adult (*P* < 0.05) and aged (*P* < 0.05) hearts in IL-36Ra–treated mice (Figure 9, C–E).

## Discussion

By 2030, it is expected that 20% of the population will be over 65 years old, and cardiovascular disease is set to account for 40% of the deaths within this age group (30). Increased age is associated with a worse prognosis post-MI and may be explained by an increased microvascular susceptibility to reperfusion injury. Therefore, consideration of the effects of aging on the postischemic heart, particularly on the smallest blood vessels of the heart, is critical. This study provides original contributions on the architecture of the aged beating heart coronary microcirculation and how its response to myocardial IR injury differs from that of younger hearts in vivo. Aging is associated with a chronic low-grade inflammatory cell presence in otherwise healthy hearts as well as a heightened thromboinflammatory response in the immediate aftermath of reperfusion. Importantly, what we believe to be a new mechanistic role is identified for the IL-36/IL-36R pathway. Our data demonstrate that IL-36 and its receptor were present in the heart and that their expression increased, particularly on coronary microvessels, with age and injury. Not only is the receptor demonstrated for the first time, as far as we are aware, to be functional in the heart, but also, importantly, its antagonism is shown to markedly reduce microcirculatory perturbations and, consequently, infarct size in adult mice. By hyperactivating innate immunity, aging has been shown to reduce the therapeutic efficacy of both pharmacological and ischemic preconditioning interventions in the heart (31, 32). Indeed, preconditioning was unable to reduce infarct size even in middle-aged rat hearts (aged 12–13 months), demonstrating that the loss of cardioprotection manifests earlier and not only in senescence (32). Therefore, it was reassuring to observe that inhibiting IL-36R signaling remained vasculoprotective even in the highly inflamed aged injured heart.

The impact of age alone on the coronary microcirculation was not negligible. Indeed, an almost 5-fold increase in neutrophil adhesion within otherwise healthy coronary capillaries was demonstrated simply as a result of the mouse age increasing from approximately 3 to 18 months. This may be linked to the observed age-related upregulation of endothelial VCAM-1 in the heart or structural and functional changes in the neutrophils themselves (33, 34). Indeed, neutrophils in aged individuals have been shown to exist in a preactivated state whereby they constitutively secrete more neutrophil elastase and ROS in close proximity to endothelium, which can lead to vascular damage and their subsequent adhesion (35). Although age-related capillary loss or rarefaction has also been described (36), no visible reduction in functional capillary density or increased vascular leakage was noted in noninjured aged hearts. Interestingly, a marked reduction in freely circulating neutrophils was observed, which may be linked to a possible decreased coronary blood flow in the aged heart (37). However, this would require further investigation. Furthermore, platelet aggregates were also visible in aged hearts and were sometimes occlusive as evidenced by the lack of circulating neutrophils passing through affected microvessels. Intravital microscopy therefore directly imaged the presence of a chronic low-grade inflammation in the aged heart but also alluded to a mildly prothrombotic state as well. These basal microcirculatory disturbances, when combined with an acute injurious insult, may create a more heightened thromboinflammatory effect in the presence of an aging comorbidity.

Indeed, this was the case in aged IR-injured hearts, where remarkable neutrophil adhesion was observed that surpassed that noted in adult injured hearts. Individual neutrophils were difficult to demarcate with clusters observed occupying the full width of the capillaries. Activated neutrophils are well known to become stiffer, which contributes to their retention specifically within capillaries that have a smaller diameter than their own (38). However, larger coronary blood vessels, which we assume were postcapillary venules (PCVs), were also delineated with adherent neutrophils, something not noted in adult injured hearts. Although neutrophil recruitment plateaued in the adult hearts, this was not the case in injured aged hearts, where neutrophils continued to be recruited. Increased platelet presence was also noted, but this was not as remarkable as the effect on neutrophils. Consequently, these thromboinflammatory occlusive events resulted in the poorest coronary microcirculatory perfusion being noted in aged injured hearts, with multiple areas devoid of flow. We believe this to be the first real-time demonstration of a rapid and devastating impact of reperfusion on the smallest blood vessels of the aged heart in vivo. This may explain the reduced myocardial tolerance to IR injury previously demonstrated to occur as early as 12 months (middle age) in mice (39).

IL-36 is typically one of the most upstream and upregulated cytokines released upon tissue injury and cellular necrosis and critical in triggering subsequent synthesis and release of a multitude of inflammatory mediators (15). This study shows that IL-36R, IL-36α, and IL-36β are constitutively expressed on vascular and nonvascular cells, albeit at very low levels, in healthy adult murine hearts. A basal expression of IL-36R is also supported by Towne and colleagues, who used quantitative PCR to demonstrate low levels in human hearts (25). Although cytokine receptor changes have not been studied extensively with age, an enhanced age-related production of cytokines such as IL-6 has previously been demonstrated (40, 41). In the current study, our data demonstrated that expression of IL-36R, IL-36α, and IL-36β increased with both age and IR injury. Expression of IL-36 agonists have been shown to increase at both the mRNA and protein level in murine kidney tissue following renal IR injury (42). Additionally, IL-36β mRNA expression increased in lung homogenates 24 hours after allergic lung inflammation (43). Our study assessed IL-36 after a much shorter reperfusion injury duration, so the full extent of IL-36 upregulation was potentially not observed.

IL-36 and IL-36R expression were observed on the majority of coronary capillaries as evidenced by colocalization with CD31^+^ endothelial cells. Moreover, it was only on microvessels that an age- and injury-related increase in cytokine and receptor expression was observed. This elevated age-related expression specifically on microvessels increases the likelihood of this signaling pathway exacerbating IR injury through targeting the coronary microcirculation in elderly patients with MI.

Immunofluorescence studies on VCECs confirmed expression of the IL-36R on endothelial cells. This was also recently shown on human umbilical vein and dermal lymphatic endothelial cells, where it was functionally important in mediating upregulation of ICAM-1/VCAM-1 and chemokine production in response to IL-36 stimulation (44). We further demonstrated that all 3 IL-36 agonists could upregulate endothelial surface expression of the IL-36 receptor. These data provide what we believe to be new insights into the fundamental biology of this cytokine. The ability of some cytokines to increase expression of their receptor is not new. Indeed, Takii and colleagues showed that IL-1 can enhance gene and surface expression of its own receptor in pulmonary fibroblast cells within 2 hours (45). Autoregulation forms a positive feedback loop that drives a strengthened activation of the signaling pathway of a given cytokine. Here, we show that IL-36 cytokines may also utilize this autoregulatory phenomenon to enhance their own activity.

Interestingly, intense IL-36/IL-36R staining was noted on the outer tunica adventitial layer of larger blood vessels. Inflammatory responses are generally considered to be initiated in an “inside-out” manner through capture of circulating leukocytes by the endothelial surface. However, growing evidence supports an “outside-in” model in which the adventitia, previously considered an inert layer that simply provides structural support, acts as an injury “sensor” within the vessel wall and subsequently directs responses to a wide array of stimuli, including ischemia. In this model, it is proposed that resident adventitial cells, such as fibroblasts, become activated and secrete inflammatory cyto/chemokines, which leads to expression of endothelial surface adhesion markers such as VCAM-1 and subsequent neutrophil recruitment to the intimal layer (46). Although recent studies have extended IL-36 and IL-36R expression to include stromal cells such as fibroblasts, further studies will be required to determine whether their presence in the adventitial vascular layer is of specific significance in mediating inflammatory responses in the heart after reperfusion injury.

A common finding in diseases where IL-36 cytokines contribute to pathology is the remarkable presence of neutrophils. Indirect evidence supporting the ability of IL-36 to recruit neutrophils has been obtained primarily from histological or flow cytometric studies in which inhibiting IL-36R signaling reduced recruitment in diseases such as psoriasis and colitis. Recent findings by Koss and colleagues pinpoint IL-36 as an early and upstream driver of acute and chronic pulmonary inflammation by promoting neutrophil recruitment and production of proinflammatory IL-1F cytokines and IL-6 (47). To directly demonstrate that IL-36 could be proinflammatory in the heart, we topically applied agonists within the center of the attached water-tight stabilizer ring. All isoforms were potently proinflammatory, something not previously shown in vivo in any organ let alone the heart. Neutrophil recruitment was observed in both coronary capillaries and PCVs but, unexpectedly, was greater in adult rather than aged hearts, which could be due to reduced neutrophil responsiveness with age (48). The inflammatory response was rapid and appeared to plateau beyond 60 minutes in adult hearts. Although it was slower and less potent in aged hearts, neutrophil recruitment did continue to rise beyond 150 minutes. It is possible that with a more prolonged imaging period, this response may have reached similar, or exceeded, maximal levels observed in adult mice. It is also possible that our observed increases in the expression of IL-36R in aged mice may be associated with a concomitant increase in circulating levels of the endogenous antiinflammatory IL-36Ra. This would act to protect the balance of IL-36 pathway signaling in aged mice by inhibiting engagement of IL-36 cytokines with their receptor. This could also explain why the same dose of topical IL-36 was unable to elicit similar or greater inflammatory responses in aged compared to adult hearts. Indeed, this has been shown to be true for IL-1Ra, where higher circulating levels are detected in elderly patients, and has been suggested to play a role in the decline in the inflammatory response with age (49).

Interestingly, we also showed that topical application of IL-36 was more potent than similar doses of topical IL-1β and TNF-α at stimulating neutrophil recruitment in both adult and aged hearts (data not presented). IL-36 also increased platelet microthrombi presence, although it is not known if this is driven through a direct response of IL-36 on platelets. It is possible that circulating platelets became trapped in vessels downstream of regions where substantial occlusive neutrophil adhesion occurred. Collectively, these studies provided a rationale for exploring the therapeutic potential of IL-36 signaling blockade to attenuate microcirculatory disturbances associated with injury.

The IL-36R is interesting in that it has 3 agonists but also 2 naturally occurring receptor antagonists, namely IL-36Ra and IL-38, which underpins the importance of careful endogenous management of the IL-36 pathway. Both competitively bind the IL-1Rrp2 component of the heterodimer receptor, preventing recruitment of the accessory coreceptor IL-1RAcP and thus inhibiting subsequent intracellular signaling (18). We tested the vasculoprotective effects of systemically injected IL-36Ra and observed a marked reduction in neutrophil recruitment in both adult and aged injured hearts. Although no antiplatelet effect was demonstrated, IL-36Ra treatment still led to a significant decrease in infarct size in both adult and aged mice. Since 1 dose of IL-36Ra was administered during the ischemic period, it is plausible that IL-36 could be targeted during PCI procedures and be therapeutically efficacious in a clinical setting.

ROS are implicated in the pathogenesis of various cardiac disorders, including MI and heart failure, and can promote the expression of endothelial adhesion molecules, such as ICAM-1 and VCAM-1, that are critical for neutrophil recruitment (50). Therefore, whether IL-36Ra mechanistically conferred vasculoprotection by limiting endothelial ROS damage and VCAM-1 expression was assessed in both adult and aged hearts. The anti-DNA/RNA antibody used in the study binds with high specificity and affinity to 8-hydroxy-2′-deoxyguanosine, 8-oxo-7,8-dihydroguanine, and 8-oxo-7,8-dihydroguanosine. These oxidative lesions serve as excellent markers for DNA and RNA damage produced specifically by ROS. Flow cytometric and immunofluorescence studies demonstrated a marked decrease in IR injury–mediated oxidative damage in the presence of the IL-36Ra. Importantly, this decrease was evident in both adult and more damaged aged hearts. Our data support the recent observation of reduced oxidative stress, measured using spectrophotometry of superoxide dismutase and malondialdehyde activity, in IL-36R–knockout rats undergoing cardiopulmonary bypass (51). However, we further detail that the antioxidant effects of IL-36Ra occur specifically on both coronary endothelial cells and cardiomyocytes. It has recently been shown that IL-36 can upregulate VCAM-1 and ICAM-1 on dermal endothelial cells in vitro and that this can be reversed by the presence of an IL-36Ra (44). However, we demonstrate the ability of IL-36Ra to decrease VCAM-1 expression in the IR-injured coronary microcirculation. Again, more importantly, this benefit was also observed in aged hearts where even basal VCAM-1 expression was high. Collectively, our data provide, we believe, novel mechanistic insights into how inhibition of IL-36/IL-36R signaling attenuates oxidative stress and VCAM-1 expression in adult and aged hearts, subsequently preventing excessive neutrophil recruitment in the coronary microcirculation, which ultimately leads to decreased infarct size postreperfusion.

### Concluding remarks.

New therapies need to be designed and optimized that are effective in improving the current poor prognosis of the aging population post-MI. We and others have recommended specific protection of the delicate coronary microcirculation from IR injury. Although studies on age-related changes of the inflammatory and immune system have gained momentum, this study is the first, as far as we know, to explore the impact of age on the coronary microcirculation in vivo both in health and postreperfusion injury. It is likely that the increased thromboinflammatory activation and microcirculatory perturbations that we have observed intravitally in the aged injured heart may inhibit the therapeutic efficacy of existing and future cardiovascular drugs in the elderly. However, our finding that IL-36Ra not only was vasculoprotective but also, importantly, remained beneficial in the setting of an age-related heightened inflammation in the coronary microvessels makes it a candidate worth pursuing clinically in elderly patients undergoing PCI for MI. In support of this is the recent work by Luo and colleagues, who demonstrated experimentally that deficiency of IL-36R protected cardiomyocytes in the setting of cardiopulmonary bypass (51).

A number of antiinflammatories, shown to be successful in experimental studies, have met with translational failure when tested in patients with MI (52). The major outcome measured in such clinical trials (and indeed experimental studies) is usually a long-term one—namely the ability to prevent post-MI remodeling, a secondary nonfatal MI, or death. Whether these antiinflammatories can also protect and keep patent the coronary microcirculation in the immediate aftermath of PCI/reperfusion has received much less interest. However, a functioning microcirculation is imperative in order to improve long-term patient outcomes. It is therefore possible that translational failure is linked to a lack of early benefit at the level of the coronary microcirculation. Since we have shown multiple microcirculatory perturbations, ultimately resulting in poor myocardial perfusion within minutes of reperfusion, it is also important that the design of an antiinflammatory therapy involves administration immediately before interventions designed to mediate reperfusion commence (e.g., PCI). However, not all clinical trials have delivered antiinflammatories prior to reperfusion, with some administered days later (53). Again, this may explain the lack of success of such compounds in clinical trials. Our data highlight a notable benefit to the coronary microcirculation and infarct size with early administration of IL-36Ra, which importantly is maintained in the presence of an aged comorbidity. This indicates that early intervention with an IL-36R inhibitor is worth considering for future clinical investigations.

## Methods

### Myocardial IR injury.

Experiments were conducted on female C57BL/6 adult (2–4 months) or aged (18–19 months) mice from Charles River in accordance with the Animals (Scientific Procedures) Act of 1986 (Project licence P552D4447). Anesthesia was induced by an intraperitoneal administration of ketamine hydrochloride (100 mg/kg) and medetomidine hydrochloride (100 mg/kg), confirmed by checking the pedal reflex every 15 minutes and maintained as required via intraperitoneal administration. Mice were intubated and ventilated with medical oxygen via a MiniVent rodent ventilator (stroke volume: 220 μL, respiratory rate: 130 breaths/min; Biochrom Ltd. Harvard Apparatus). The carotid artery was cannulated to facilitate infusion of antibodies, dyes, saline, and IL-36Ra. IR injury was induced by ligating the left anterior descending (LAD) artery for 45 minutes and reperfusion allowed to proceed for 2 hours (tissue analysis), 2.5 hours (intravital observations), or 4 hours (infarct measurement). Sham surgery involved the same procedure but without LAD artery ligation. At the end of experiments, mice were euthanized by cervical dislocation, and euthanasia was confirmed by ensuring cessation of the circulation by making an incision in the carotid artery.

### Intravital imaging of the beating heart coronary microcirculation.

Real-time intravital observations were performed as previously described (28). Briefly, a 3D-printed stabilizer was permanently fixed to the left ventricle downstream of the ligation site (Figure 4, A–C). To simultaneously image endogenous neutrophils and platelets, PE anti–mouse Ly-6G (BioLegend clone RB6-8C5) and APC anti–mouse CD41 (BioLegend clone MWRed30) were injected 5 minutes prior to reperfusion. Intravital imaging was performed using a microscope (BX61WI, Olympus) equipped with a Nipkow spinning disk confocal head (Yokogawa CSU) and an Evolve EMCCD camera (Photometrics). The first 2-minute capture was performed at 15 minutes postreperfusion, followed by 2-minute captures every 15 minutes of the same area for 2.5 hours. In separate mice, FITC-BSA (Sigma) was injected at the end of a 2.5-hour reperfusion period to investigate overall vascular perfusion and functional capillary density.

For some studies, recombinant mouse IL-36Ra (15 μg/mouse; Novus Biologicals) was injected intra-arterially at both 10 minutes prereperfusion and 60 minutes postreperfusion. One of the critical factors that limits the clinical success of previously tested antiinflammatory drugs is the time of intervention. Given the rapid development of microvascular no-reflow, the first hours, if not minutes, following reperfusion are critical. Our treatment strategy focused on introducing the antagonist during the ischemic phase to establish an effective circulating concentration to dampen the initial reperfusion-associated inflammatory response and maintain microvascular patency. The ability of IL-36 cytokines to directly mediate thromboinflammatory events in vivo was also assessed. After placing the stabilizer on the healthy heart, 20 μL of recombinant mouse IL-36 cytokine (α, β, or γ; 200 ng/mL) or PBS was topically applied to the heart surface within the water-tight stabilizer ring for 15 minutes and a 2-minute video recorded. This was replaced with fresh cytokine/PBS for 15 minutes and the process repeated 10 times for a total duration of 2.5 hours.

Data were captured, stored, and analyzed using Slidebook 6 software (Intelligent Imaging Innovations). Free-flowing neutrophils, which passed through the coronary microcirculation without making adhesive interactions, were counted manually over the 2-minute recorded capture. To analyze adherent neutrophil and platelet presence, captured videos were subjected to postacquisition image repair using in-house–designed software (*Tify*) in which out-of-focus frames were removed (54). Neutrophils and platelet aggregates/microthrombi were then quantitated by placing a mask around PE-Ly6G^+^ and APC-CD41^+^ areas, respectively. Integrated fluorescence density, which took into account size and fluorescence intensity, was then calculated using ImageJ (NIH).

### Multiphoton imaging of heart sections.

Intravital imaging captured microvascular events from the surface of the beating heart with a depth of approximately 50–60 μm. To determine whether these events were mirrored throughout the thickness of the ventricular wall, multiphoton microscopy was performed on hearts harvested at the end of intravital experimentation. The left ventricle was sectioned into 4 sections of 300 μm using a tissue vibratome (Campden Instruments Limited) and imaged from the epicardial through to the endocardial end using a multiphoton microscope (FVMPE-RS Olympus). *Z*-stacks from the 4 layers were rendered to form 3D stack images, which were processed and displayed using ImageJ (Figure 4, E and F). The presence of neutrophils was analyzed as the sum fluorescence intensity for each section (ImageJ).

### Immunohistochemistry analysis of IL-36 cytokines and IL-36R.

Ten-micrometer sections of frozen heart tissue were incubated at room temperature with primary anti–IL-36R, anti–IL-36α, anti–IL-36β, or IgG control antibodies (1:100 dilution; R&D Systems, polyclonal) and a secondary donkey anti–goat Alexa Fluor 488 antibody (1:100 dilution; Abcam, polyclonal). Sections were also incubated with a PE anti–mouse CD31 antibody (1:100 dilution, BioLegend, clone 390), an Alexa Fluor 647 anti–mouse CD106/VCAM-1 antibody (1:100 dilution, BioLegend, clone 429), or an anti-DNA/RNA damage antibody to detect oxidative damage (1:100 dilution, Abcam, clone 153A). Images were captured using an EVOS FL (Thermo Fisher Scientific) or multiphoton microscope (FVMPE-RS, Olympus). ImageJ was used to quantify the MFI of each image with additional analysis of MFI on regions containing only coronary capillaries or a large blood vessel.

### Western blotting analysis.

Total protein was extracted from harvested hearts using RIPA buffer and homogenization with beads. Lysates were normalized using a BCA protein assay kit (Thermo Fisher Scientific) to 2 mg/mL. Samples were run on an SDS-PAGE gel and then transferred onto a nitrocellulose membrane before being blocked for unspecific binding with 5% nonfat dried milk. The membrane was then incubated overnight at 4°C with the primary antibody for IL-36R (1:200 dilution) followed by incubation with the secondary antibody conjugated to Alexa Fluor 488 (1:1000 dilution). After washing, protein bands were visualized using a fluorescence detection system (ChemiDoc, Bio-Rad) and MFI was determined.

### Flow cytometric analysis of endothelial and cardiomyocyte oxidative stress.

Harvested adult and aged hearts were manually minced, added to 0.1% collagenase, and rotated in an incubator at 37°C for 15 minutes. The supernatant was removed, and the digestion process was repeated 2 times. The pellet was then centrifuged at 10000 rpm at 4°C for 10 minutes in the presence of ACK and MACS buffer to lyse red blood cells and stop enzymatic activity, respectively. The pellet was added to 20 mL media and run several times through a 70 μm strainer. Cells were then incubated with an anti-DNA/RNA damage antibody to detect oxidative damage (1:100 dilution, Abcam, clone 153A), anti–IL-36R antibody to detect IL-36R expression (R&D Systems, polyclonal; Alexa Fluor 647 secondary, BioLegend, polyclonal; both at 1:100 dilution), anti-CD31 antibody to label endothelial cells (1:100 dilution, BioLegend, clone 390), anti-cTnT antibody to label cardiomyocytes (1:100 dilution, Miltenyi Biotec, clone REA400), Zombie to detect dead cells (1:500 dilution, BioLegend), and appropriate IgG controls (polyclonal). Cells were then fixed using 4% formalin for 10 minutes and washed with Dulbecco’s PBS. Acquisition of cells was performed using a CyAn ADP (Beckman Coulter), and data analysis was performed using Summit 4.3 software (Beckman Coulter). For each sample, 250,000 events were captured and used in the analysis.

### Endothelial cell IL-36R expression analysis in vitro.

Immortalized murine VCECs were grown to confluence and stimulated for 4 hours with experimental media (vehicle control); recombinant mouse IL-36α, β, or γ (3, 30, or 300 ng/mL; R&D Systems); or recombinant mouse TNF-α (3, 30, or 300 ng/mL; Boster Biological Technology). Cells were then formalin-fixed and incubated overnight with a primary antibody against IL-36R (1:100 dilution), followed by incubation with a secondary antibody (1:100 dilution) and Hoechst 33342 dye (Thermo Fisher Scientific). Images were captured using a multiphoton microscope and MFI was determined (ImageJ).

### Myocardial infarct size analysis.

The LAD artery was religated 4 hours after reperfusion, and 0.5% Evans blue dye (Sigma) was infused via the carotid cannula to identify the AAR. The mouse was then sacrificed, and the harvested heart was cut into sequential slices and incubated with TTC (Sigma). Sections were imaged using a stereomicroscope, and analysis was performed using ImageJ to quantitate the infarct size (TTC-negative white regions) as a percentage of the AAR (TTC-positive red regions/Evans blue–negative).

### Data availability.

The authors confirm that the data supporting the findings of this study are available within the article and its supplemental materials. Raw data supporting the findings of this study are available from the corresponding author, NK, on request.

### Statistics.

All statistical analysis was performed using GraphPad 7.0 software (GraphPad Software Inc.). Multiple comparisons between 3 or more groups were performed by 1-way ANOVA, followed by a Tukey’s post hoc test. For experiments that followed a time course, the AUC was also calculated and used for subsequent analysis as a summation of the entire period. All data are presented as mean ± SEM with statistical significance defined when *P* < 0.05.

### Study approval.

Experiments were conducted on mice in accordance with the Animals (Scientific Procedures) Act of 1986 (Project licence P552D4447 from Home Office, London, United Kingdom).

## Author contributions

JEA acquired, analyzed, and interpreted the data; drafted the work; and approved the submitted version. DPJK analyzed and interpreted the data and approved the submitted version. MRR provided material and approved the submitted version. CJW provided materials and approved the submitted version. NED approved the submitted version. NK obtained the funding, designed the experiments, interpreted the data, drafted the work, and approved the submitted version. All authors agreed to be personally accountable for contributions and to ensure that questions related to the accuracy or integrity of any part of the work are appropriately investigated and resolved, with the resolution documented in the literature.

## Supplementary Material

Supplemental video 1

Supplemental video 2

Supplemental video 3

## Version 1 02/03/2022

In-Press Preview

## Version 2 03/08/2022

Electronic publication
